# Association between the FAS/FASL Variants and Risk of Male Infertility in Asian Populations; A Systematic Review and Meta-Analysis

**DOI:** 10.3390/medicina55060247

**Published:** 2019-06-05

**Authors:** Rezvan Asgari, Kamran Mansouri, Mitra Bakhtiari, Hadi Mozafari, Shiva Roshankhah

**Affiliations:** 1 Medical Biology Research Center (MBRC), Kermanshah University of Medical Sciences, Kermanshah 6714415185, Iran; R.asgari33@gmail.com (R.A.); kmansouri@kums.ac.ir (K.M.); 2 Fertility and Infertility Research Center, Health Technology Institute, Kermanshah University of Medical Sciences, Kermanshah 6714869914, Iran; sh.roshankhah@kums.ac.ir

**Keywords:** FAS, FASL, polymorphism, male infertility, meta-analysis

## Abstract

*Background and Objectives*: Studies suggest that FAS/FASL polymorphisms are associated with male infertility; however, their results are still inconclusive. Therefore, this systematic review and meta-analysis aimed to summarize and clarify the overall association of FAS/FASL polymorphisms and risk of male infertility. *Materials and Methods*: Our search was conducted on the databases of Science Direct, PubMed and Google Scholar. For performing the meta-analysis, pooled odds ratio (OR) values with 95% confidence interval (CI) was applied in order to analyze the strength of association between the FAS/FASL polymorphisms and risk of male infertility. A total of seven relevant studies published up to September 2018 were considered. *Results*: FASL-844C/T genotype results of 559 patients and 623 healthy individuals were included in our study. For FAS-670A/G genotype effect, 751 patients and 821 healthy individuals were explored. Results showed that all analysis models including dominant, recessive and allelic models of FASL-844C/T and FAS-670A/G polymorphism had no significant effect on infertility in men (*p* > 0.05 and *p* > 0.05, respectively). According to sensitivity analysis, our results were stable. *Conclusion*: We demonstrated that FAS/FASL polymorphisms might not be an effective factor on male reproductive health. For precise determination of FAS/FASL polymorphisms effects on male infertility, large-scale case-control studies should be performed.

## 1. Introduction

Recently, infertility has been increasing dramatically and it has become a major public health problem worldwide affecting about 15% of couples [1]. Therefore, new diagnostic markers seem to be of high value for detection and prevention of infertility. Male infertility is a biological process still not completely understood. Despite the significant progress in male infertility diagnosis, there are many genetic factors that remain unknown. According to the review of the literature, correlation of genetic factors with environmental agents might contribute to infertility in men [2,3,4,5]. A strong association has been reported between defects in apoptosis-related genes with many human diseases, including male infertility [6,7], for which genetic polymorphisms are of influence. Thus, several studies have focused on the association of the variants in apoptosis-related genes and the male infertility susceptibility [7,8].

Because of the small sample sizes and various ethnic backgrounds in some of the already published studies, the existing results are contradictory. Hence, all qualified data needs to be retrieved in order to investigate whether genetic polymorphisms are associated with an increased risk of male infertility. During sperm maturation, several of the dysfunctional sperms are destroyed for which purpose apoptosis, both internal and external pathways, is the main event [9]. On the other hand, apoptosis regulation of germ cells during spermatogenesis is quite critical. Accordingly, the main focus of different studies has been placed on the FAS/FASL system as a core mean of apoptotic signal transmission leading to cell death [10,11]. Some functional polymorphisms in the promoter region of these two genes include -670A/G in the *FAS* gene and -844C/T in the *FASL* gene, which might be related to the increased risk of various diseases such as cancer and infertility [7,12,13]. The polymorphism of FAS-670A/G is the most common single-nucleotide polymorphism (SNP) in *FAS* gene’s promoter region. Substitution of a G for A at -670 bp causes a mutation in the binding site of STAT1 transcription factor which leads to a decreased transcription level of this gene [14]. The FASL-844C/T polymorphism located in the promoter region of *FASL* gene is associated with alterations in the binding motif of CAAT/enhancer-binding protein β element. A T for C substitution at the position of -844 on the other hand, may affect the expression levels of *FASL* gene [15].

Several studies have reported an association between the polymorphisms of FAS-670A/G and FASL-844C/T and male infertility risk [8,16,17]. Currently, there are no systematic reviews and meta-analysis data available on the relationship of these important polymorphisms and susceptibility to infertility in men. Therefore, this study evaluated the association between the polymorphisms of FAS-670A/G and FASL-844C/T with male infertility by performing a systematic review and meta-analysis based on available independent studies.

## 2. Materials and Methods

### 2.1. Literature Search

Results from different studies were collected from electronic databases of Science Direct, PubMed and Google Scholar (updated March 2018). We used the combination of the key words as follows “polymorphism/genotype/variant”, “FAS/CD95/TNFSF6/APO-1”, “FASL/CD95L” and “male infertility/azoospermia/oligozoospermia/teratospermia”. Study selection criteria were: (1) studies of case-control; (2) studies on human beings, (3) investigation of relationship between the polymorphisms of FAS and/or FASL and male infertility risk, (4) presentation of sufficient data on the sample size, odds ratio (OR) with 95% confidence interval (CI).The exclusion criteria were those studies with no raw data for OR calculation with corresponding 95% CI, case-only, conference abstracts, editorials, case reports and review articles (including meta-analysis).

### 2.2. Data Extraction

Data was extracted from all qualified publications by two authors independently (R. Asgari and M. Bakhtiari) according to the above-listed inclusion and exclusion criteria. The following characteristics were extracted from enrolled studies: the first author’s name, year of publication, country, population ethnicity, genotype frequency for groups of cases and controls, sites of polymorphisms, minor allele frequency (MAF) and *p*-value for Hardy–Weinberg equilibrium (HWE) as listed in Table 1, Table 2 and Table 3.

### 2.3. Statistical Analysis

Hardy–Weinberg equilibrium and MAF were calculated manually. The strength of the association between FAS-670 and FASL-844 polymorphisms with the risk of male infertility was estimated by pooled OR with 95% CI using Der Simonian and Laird (random effects model) or Mantel-Haenszel method (fixed effects model) according to heterogeneity evaluation. Publication bias was identified by Begg’s test and Egger’s test. The level of *p* < 0.05 was considered statistically significant. All calculations were performed by Medcalc and CMA 2 software. Finally, we determined the statistical power for all analysis models by PS (Power and Sample Size Calculations), version 3.1.2.

## 3. Results

### 3.1. Characteristics of Eligible Studies

**Figure 1:**
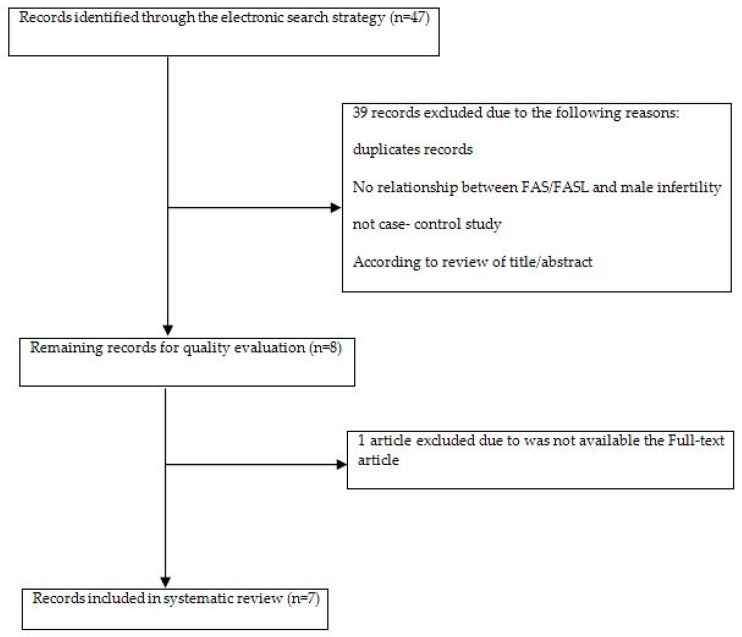
Flow diagram of the search and study selection process according to inclusion and exclusion criteria. FASL: FAS Ligand.

A total of seven relevant publications, published between 2007 and 2017, were included in the present study. Figure 1 shows the trial flow diagram for process of study selection. The studies included 1993 patients and controls and were mainly conducted on Asian populations. Similarly, for the evaluation of genetic variants, genomic DNA had been extracted from the peripheral blood samples and PCR-RFLP )polymerase chain reaction-restriction fragment length polymorphism) technique had been used in all the studies. The main characteristics of included studies for FAS/FASL polymorphisms are listed in Table 1. In addition, the data on the relationship between these polymorphisms and male infertility is illustrated in Table 2 and Table 3.

### 3.2. Association Between FAS/FASL Polymorphisms and Male Infertility

As shown in Table 2 and Table 3, only one study demonstrates a significant association between infertility inpatients and FAS-670A/G polymorphism (*p*-value < 0.05) [18]. Additionally, the studies report conflicting results on the association of FASL-844C/T polymorphism with male infertility risk. The results of three studies by Wang et al., Hassan et al. and Asgari et al. [8,17,18] suggested that FASL-844C/T polymorphism has a significant association with male infertility showing that it may serve as a factor of influence. While Hassan et al. obtained similar results studying Iraqi patients; Jaiswal et al. [19] demonstrated no such association in Indian population. Likewise, other studies [8,16,17,20,21] did not achieve any significant difference between infertility patients and controls.

### 3.3. Results of the Meta-Analysis

**Figure 2:**
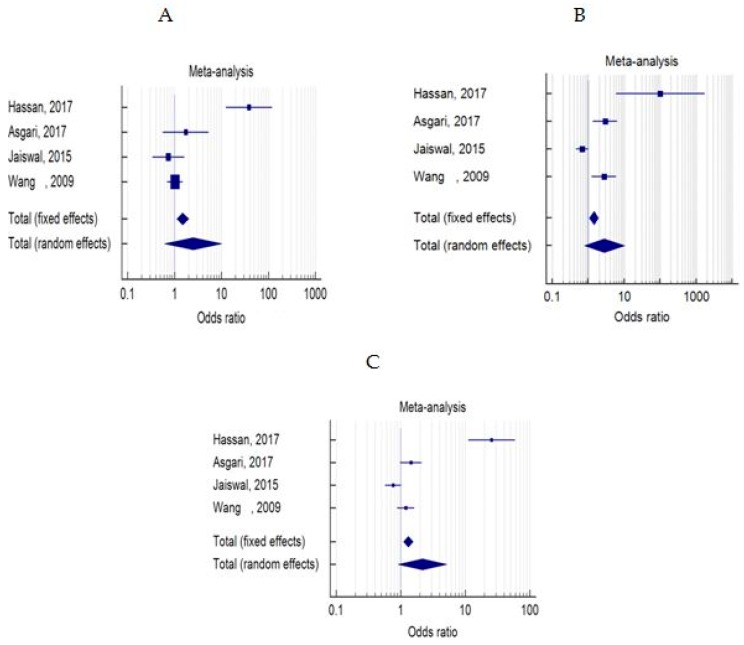
Forest plots of odds ratios for the association between FASL-844C/T and male infertility in models of dominant, recessive and allelic ((**A**–**C**) for models of dominant, recessive and allelic, respectively).

**Figure 3:**
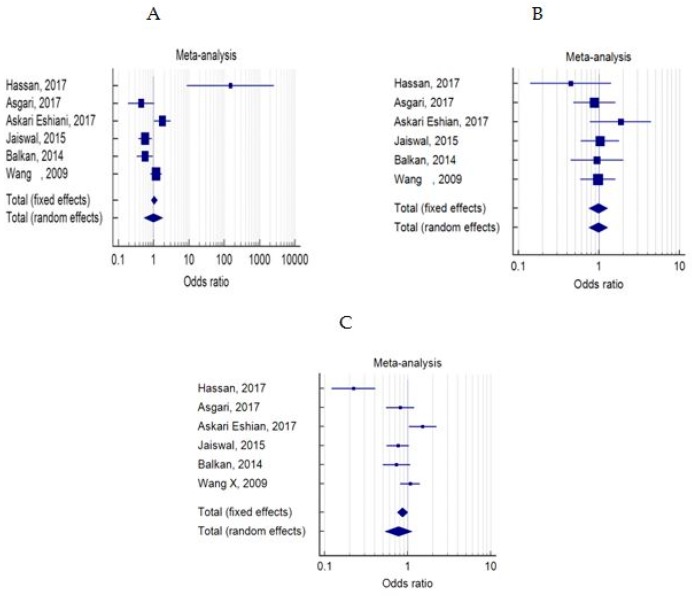
Forest plots of odds ratios for the association between FAS-670A/G and male infertility in models of dominant, recessive and allelic ((**A**–**C**) for models of dominant, recessive and allelic, respectively).

The distribution of FASL-844 variants in models of dominant, recessive and allelic was not significantly different between case and control groups (Table 4, Table 5 and Table 6 and Figure 2A–C). Likewise, we did not find any significant relevance between FAS-670A/G polymorphism and male infertility in dominant, recessive or allelic models (Table 7, Table 8 and Table 9 and Figure 3A–C). Moreover, no significant difference was detected between the wild genotype (CC or AA) and heterozygote (CT or AG) or homozygote (TT or GG) types of mutations in both studied polymorphisms in case and control groups (data not shown).

### 3.4. Results of Heterogeneity Test

For both FASL-844C/T and FAS-670A/G polymorphisms significant heterogeneity was observed in dominant, recessive and allelic models (except for the recessive model of FAS-670A/G polymorphism) in case and control groups (Table 4, Table 5, Table 6, Table 7, Table 8 and Table 9). This rate of heterogeneity was mainly presented by one study [16]. Therefore, levels of *p*-value and OR were calculated according to random effects except for the recessive model of FAS-670A/G polymorphism (which *p*-value and OR were calculated according to fixed effects).

### 3.5. Sensitivity Analysis and Publication Bias

**Figure 4:**
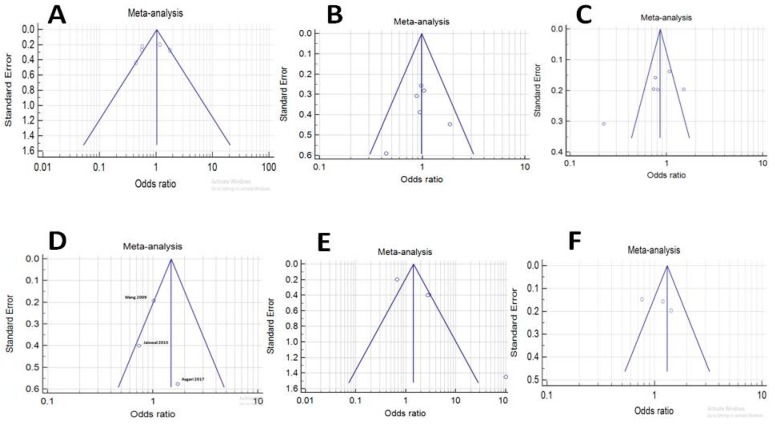
Bias of literature for FASL-844C/T and FAS-670A/G was tested by Begg’s funnel plot and Egger’s test results are depicted for three different analysis models of FAS-670A/G (**A**): dominant; (**B**): recessive; (**C**): allelic and FASL-844C/T (**D**): dominant; (**E**): recessive; (**F**): allelic.

Sensitivity analysis was carried out using accumulative analysis and removing the one mentioned study to evaluate the effect of that individual study on the overall OR. This analysis indicated that ORs or *p*-value were not altered for FASL-844C/T polymorphism and our results were stable. Funnel plots, Begg’s test and Egger’s test were performed to assess publication bias, of which the results are illustrated in Table 10 and Figure 4A–F. According to Egger’s test, two models of FASL-844C/T polymorphism (recessive and allelic model), and according to Begg’s test, the dominant model of FASL-844C/T polymorphism, had publication bias. Based on both tests, however, all models of analysis for FAS-670A/G polymorphism did not show any publication bias.

## 4. Discussion

For the first time in the present meta-analysis, we studied the genetic relevance between FAS-670A/G and FASL-844C/T polymorphisms of different models and the risk of male infertility. Generally, the current study involved 955 infertile men and 1038 fertile men (as controls) from the populations of Iran, China, India, Turkey and Iraq. Herein, we investigated the role of these variants in male infertility under genetic models of dominant, recessive and allelic. Our combined results failed to find any statistically significant difference, mutation- or genetic-wise between FAS and FASL variants with male infertility (*p* > 0.05). This result was consistent with the data from certain studies included herein. However, taking a closer look at the data, in the allelic model, the results were almost statistically significant (*p* = 0.08 and *p* = 0.09 for FASL-844C/T and FAS-680A/G, respectively). This is as good a point as any to mention that the difference between *p*-value cutoff and our *p*-values might have been avoided upon a larger number of the population studied.

Since different studies have reported the importance of *FAS* and *FASL* genes in spermatogenesis, it is plausible that polymorphisms of these genes could have a potential influence on the expression of FAS and/or FASL and, therefore, might be related to male infertility [7,8,15,22]. The FAS/FASL interaction triggers the death signal cascade which subsequently induces apoptosis in many cell types and tissues including testis [6]. According to review of the literature, down-regulation of FAS expression and/or up-regulation of FASL expression have been indicated in many types of disorders and diseases such as infertility [15,23]. In 1999, the role of the FAS-mediated pathway in failed apoptosis and its relationship with spermatogenesis process were explained by Sakkas et al. [23]. Meanwhile, they showed that men with normal semen parameters had low levels of *FAS* expression compared to those with abnormal semen parameters. Likewise, these findings were confirmed later using the immune activity assay [24]. For the first time, a study by Ji et al. reported that FAS-670A/G polymorphism might associate with apoptotic mechanism in germ cells and semen quality. Indeed, this study showed that men with GG genotype had a relatively low rate of apoptosis, poor sperm motility, and reduced sperm concentration in ejaculated semen compared to individuals carrying AA genotype [7].

Additionally, the study by Wang et al. showed that FASL-844C/T polymorphism was significantly correlated with an increased risk of idiopathic azoospermia. In other words, it might be a genetic predisposing agent of idiopathic azoospermia or severe oligozoospermia among Han Chinese men. However, the authors found no association between the polymorphism of FAS-670 A/G and idiopathic male infertility [8]. In consistent with this report, Hassan et al. reported a significant relevance between FASL-844T allele and risk of male infertility [18]. In addition, Balkan et al. demonstrated that GG and AA homozygous genotypes in polymorphism of FAS-670A/G was correlated with high potential of idiopathic azoospermia. Moreover, they indicated that heterozygous AG genotype was significantly lower in patients compared to controls; this in turn shows that heterozygous genotypes in this variant have possibly had a protective effect against idiopathic azoospermia. In fact, these results confirm that FAS-670 A/G polymorphism is a risk factor predisposing the male sex to infertility in the Turkish population [16]. The present study, on the other hand, is the first meta-analysis to examine and study the possible association of FAS-670A/G polymorphism with male infertility; the results proved this variation to not affect male fertility. To confirm our results, the investigation of a larger number of subjects in a case–control study would be essential. As Asgari et al. have illustrated before, the FASL-844C/T polymorphism is associated with the idiopathic azoospermia. Thus, this variation could in fact be considered a risk factor for male infertility in population of Western Iran. Nevertheless, they found no association of FAS-670A/G polymorphism with idiopathic azoospermia in this population [17]. In contrast, in two studies by Jaiswal et al. on the Indian population, no significant association was found between polymorphisms of FAS -670A/G and FASL-844C/T and male infertility [19,21]. Nevertheless, Hassan et al. have reported that FAS-670A/G polymorphism is significantly linked to male infertility [18]. Due to the inconsistency of these results, the present study was carried out to address the mentioned ambiguities and provide more evidence on the association of these two variants with risk of male infertility.

We believe that since the number of our included studies was small, the results from the correlation of FAS-670A/G polymorphism and male infertility should be interpreted with caution. Therefore, in order to achieve a clear conclusion and a better determination of correlation between these polymorphisms and the risk of male infertility, major gene–gene and gene-environment interaction researches in various populations and great sample sizes are necessary. Moreover, studied subjects in the present study were from Middle-Eastern (Turkey, Iraqi and Iranian) and Asian (Indian and Chinese) patients. Since no such study has been carried out in other to parts of the world, we were not able to perform a subgroup analysis stratified by ethnicity. Another limitation to our study was the data insufficiency of the included studies. Therefore, it was not feasible to carry out subgroup analysis on smoking, alcoholism and age categories.

## 5. Conclusions

Our meta-analysis showed that FAS/FASL polymorphisms might is not associated with the risk of male infertility. However, polymorphism of FASL-844C/T might affect fertility potential in men. To confirm our results and determining the biological roles of these functional polymorphisms in this condition, more extensive and comprehensive case-control studies in a larger population should be designed.

## Figures and Tables

**Table 1:** Main properties of studies included in this systematic review and meta-analysis.

First Author	Year	Age: Controls Patients	Ethnicity	Number of Participants (Total/Control/Case)	Detected Sample	FAS/FASL
Askari Eshiani RA	2017	25–40	Iranian	234/102/132	Blood	-670A/G
Asgari R	2017	32.41 ± 6.43 33.91 ± 7.43	Iranian	212/110/102	Blood	-670A/G -844C/T
Wang W	2009	-	Chinese	449/246/203	Blood	-670A/G -844C/T
Balkan M	2014	37.8 ± 7.6 31.3 ± 5.5	Turkish	233/125/108	Blood	-670A/G
Jaiswal D	2012	Matched 32.0 ± 4.8	Indian	344/188/156	Blood	-670A/G
Jaiswal D	2015	Matched 32.0 ± 4.8	Indian	421/217/204	Blood	-844C/T
Hassan GM	2017	-	Iraqi	100/50/50	Blood	-670A/G -844C/T

**Table 2:** Summary results of association between FAS-670A/G polymorphism and idiopathic azoospermia.

First Author	Year	FAS Polymorphism	Allele/Genotype -670A/G: (A-G)/(AA-AG-GG)	MAF	^P^HWE	OR	95% CI	*p*-Value
Askari Eshiani RA	2017	-670A/G	Controls: (140-64)/(46-48-8)	0.313	0.348	1.76	1.03–3	0.07
			Patients: (156-108)/(42-72-18)		0.140			
Asgari R	2017	-670A/G	Controls: (88-132)/(10-68-32)	0.6	0.002	0.44	0.18–1.05	NS
			Patients: (92-112)/(17-58-27)		0.134			
Wang W	2009	-670A/G	Controls: (305-187)/(100-105-41)	0.38	0.139	1.17	0.78–1.75	NS
			Patients: (245-161)/(75-95-33)		0.751			
Balkan M	2014	-670A/G	Controls: (151-99)/(43-65-17)	0.396	0.330	0.56	0.33–0.95	NS
			Patients: (146-70)/(52-42-14)		0.243			
Jaiswal D	2012	-670A/G	Controls: (218-158)/(64-90-34)	0.42	0.809	1.03	0.59–1.78	NS
			Patients: (201-111)/(74-53-29)		0.0012			
Hassan GM	2017	-670A/G	Controls: (40-60)/(0-40-10)	0.90	0.0000	150.2638.5	8.76–2575	<0.001
			Patients: (75-25)/(30-15-5)		0.157			<0.001

^P^HWE: *p*-value for Hardy-Weinberg Equilibrium.

**Table 3:** Summary results of association between FASL-844C/T polymorphism and idiopathic azoospermia.

First Author	Year	FASL Polymorphism	Allele/Genotype -844C/T: (C-T)/(CC-CT-TT)	MAF	^P^HWE	OR	95% CI	*p*-Value
Asgari R	2017	-844C/T	Controls: (108-112)/(9-90-11)	0.509	0.0000	2.02	1.05–3.88	0.02
			Patients: (82-122)/(5-72-25)		0.0000			
Wang W	2009	-844C/T	Controls: (380-112)/(144-92-10)	0.227	0.319	2.72	1.25–5.93	0.024
			Patients: (300-106)/(118-64-21)		0.0091			
Jaiswal D	2015	-844C/T	Controls: (128-306)/(12-104-101)	0.705	0.0247	0.73	0.33–1.61	NS
			Patients: (144-264)/(15-114-75)		0.0014			
Hassan GM	2017	-844C/T	Controls: (92-8)/(42-8-0)	0.08	0.538	638.5	12.3–120.3	<0.001
			Patients: (31-69)/(6-19-25)		0.429			

^P^HWE: *p*-value for Hardy-Weinberg Equilibrium.

**Table 4:** Meta-analysis of the association between FASL-844C/T and male infertility in dominant model. D–L, DerSimonian and Laird method, CI, confidence interval.

First Author	Experimental Events/ Total (Case)	Control Events/Total (Control)	Weight%	Odds Ratio D-L, Random, 95% CI
Hassan et al.	44/50	8/50	23.52	38.5(12.31–120.36)
Asgari et al.	97/102	101/110	23.59	1.73(0.56–5.34)
Jaiswal et al.	189/204	205/217	25.61	0.73(0.33–1.61)
Wang et al.	85/203	102/246	27.28	1.02(0.70–1.48)
Total	559	623	100	2.49(0.61–10.16)

Heterogeneity: *p* < 0.0001; I^2^ = 92.19% (95% CI: 83.2–96.37); Statistical Power = 100%, *p* = 0.202.

**Table 5:** Meta-analysis of the association between FASL-844C/T and male infertility in recessive model. CI, confidence interval.

First Author	Experimental Events/Total (Case)	Control Events/Total (Control)	Weight%	Odds Ratio D-L, Random, 95% CI
Hassan et al.	25/50	0/50	12.27	101(5.9–1727.16)
Asgari et al.	25/102	11/110	28.47	2.92(1.35–6.30)
Jaiswal et al.	75/204	101/217	30.86	0.66(0.45–0.98)
Wang et al.	21/203	10/246	28.40	2.72(1.25–5.92)
Total	559	623	100	2.80(0.78–9.98)

Heterogeneity: *p* < 0.0001; I^2^ = 89.74% (95% CI: 76.61–95.50); Statistical Power = 100%, *p* = 0.111.

**Table 6:** Meta-analysis of the association between FASL-844C/T and male infertility in allelic model. CI, confidence interval.

First Author	Experimental Events/Total (Case)	Control Events/Total (Control)	Weight%	Odds Ratio D-L, Random, 95% CI
Hassan et al.	69/100	8/100	21.71	25.59(11.07–59.14)
Asgari et al.	122/204	112/220	25.75	1.43(0.97–2.1)
Jaiswal et al.	264/408	306/434	26.32	0.76(0.57–1.02)
Wang et al.	106/406	112/492	26.23	1.19(0.88–1.62)
Total	1118	1246	100	2.16(0.9–5.2)

Heterogeneity: *p* < 0.0001; I^2^ = 95.17% (95% CI: 90.59–97.52); Statistical Power = 100%, *p* = 0.08.

**Table 7:** Meta-analysis of the association between FAS-670A/G and male infertility in dominant model. CI, confidence interval.

First Author	Experimental Events/Total (Case)	Control Events/Total (Control)	Weight%	Odds Ratio D-L, Random, 95% CI
Hassan et al.	50/50	20/50	3.95	150.26(8.76–2575)
Asgari et al.	85/102	101/110	15.83	0.44(0.18–1.05)
Askari Eshtiani et al.	90/132	56/102	19.42	1.76(1.03–3)
Jaiswal et al.	82/156	124/188	20.41	0.57(0.37–0.88)
Balkan et al.	56/108	82/125	19.49	0.56(0.33–0.95)
Wang et al.	128/203	146/246	20.90	1.16(0.79–1.71)
Total	751	821	100	0.98(0.53–1.83)

Heterogeneity: *p* < 0.0001; I^2^ = 84.28% (95% CI: 67.42–92.42); Statistical Power = 5.4%, *p* = 0.96.

**Table 8:** Meta-analysis of the association between FAS-670A/G and male infertility in recessive model. M-H, Mantel-Haenszel method; CI, confidence interval.

First Author	Experimental Events/Total (Case)	Control Events/Total (Control)	Weight%	Odds Ratio M-H, Random, 95% CI
Hassan et al.	5/50	10/50	5.42	0.44(0.14–1.41)
Asgari et al.	27/102	32/110	19.91	0.87(0.48–1.6)
Askari Wshtiani et al.	18/132	8/102	9.4	1.85(0.77–4.45)
Jaiswal et al.	29/156	34/188	24.02	1.03(0.59–1.79)
Balkan et al.	14/108	17/125	12.52	0.94(0.44–2.02)
Wang et al.	33/203	41/246	28.73	0.97(0.58–1.60)
Total	751	821	100	0.98(0.75–1.28)

Heterogeneity: *p* = 0.54; I^2^ = 0.00% (95% CI: 0.00–69.30); Statistical Power = 5.2%, *p* = 0.88.

**Table 9:** Meta-analysis of the association between FAS-670A/G and male infertility in allelic model. CI, confidence interval.

First Author	Experimental Events/Total (Case)	Control Events/Total (Control)	Weight %	Odds Ratio D-L, Random, 95% CI
Hassan et al.	25/100	60/100	13.35	0.22(0.12–0.40)
Asgari et al.	112/204	132/220	16.76	0.81(0.55–1.19)
Askari Eshtiani et al.	108/264	64/204	16.78	1.51(1.03–2.22)
Jaiswal et al.	111/312	158/376	17.88	0.76(0.55–1.07)
Balkan et al.	70/216	99/250	16.82	0.73(0.49–1.07)
Wang et al.	161/406	187/492	18.41	1.07(0.81–1.40)
Total	1502	1642	100	0.77(0.53–1.13)

Heterogeneity: *p* < 0.0001; I^2^ = 84.14% (95% CI: 67.08–92.36); Statistical Power = 94.7%, *p* = 0.09.

**Table 10:** Egger’s test and Begg’s test results for funnel plot asymmetries of FAS/FASL polymorphisms.

Polymorphism (Model)	Egger’s Test *p*-Value	95% CI	Begg’s Test *p*-Value
FASL-844C/T (Dominant)	0.19	−12.10–20.37	0.02
FASL-844C/T (Recessive)	0.02	−0.09–10.09	0.08
FASL-844C/T (Allelic)	0.01	0.63–8.21	0.08
FAS-670A/G (Dominant)	0.21	−4.79–9.28	0.09
FAS-670A/G (Recessive)	0.36	−4.86–3.74	0.28
FAS-670A/G (Allelic)	0.15	−17.27–3.81	0.34
